# Supplementary material for: Attention network modulation via tRNS correlates with attention gain
Source: eLife. 2021 Nov 26;10:e63782. doi: 10.7554/eLife.63782 (PMC8626087; doi:10.7554/eLife.63782)
Supplement: Transparent reporting form [file elife-63782-transrepform1.docx]

***eLife’s* transparent reporting form**

We encourage authors to provide detailed information *within their submission* to facilitate the interpretation and replication of experiments. Authors can upload supporting documentation to indicate the use of appropriate reporting guidelines for health-related research (see [EQUATOR Network](http://www.equator-network.org/%20)), life science research (see the [BioSharing Information Resource](https://biosharing.org/)), or the [ARRIVE guidelines](http://www.plosbiology.org/article/info:doi/10.1371/journal.pbio.1000412) for reporting work involving animal research. Where applicable, authors should refer to any relevant reporting standards documents in this form.

If you have any questions, please consult our Journal Policies and/or contact us: [editorial@elifesciences.org](mailto:editorial@elifesciences.org).

**Sample-size estimation**

- You should state whether an appropriate sample size was computed when the study was being designed
- You should state the statistical method of sample size computation and any required assumptions
- If no explicit power analysis was used, you should describe how you decided what sample (replicate) size (number) to use

Please outline where this information can be found within the submission (e.g., sections or figure legends), or explain why this information doesn’t apply to your submission:

An explicit power analysis was not performed. However, the sample size was based on previously published studies that used neurostimulation procedures coupled with learning and/or neuroimaging techniques and has a similar (or fewer) number of participants.

In particular, Helfrich and colleagues (Plos Biology, 2014) used a combination of high-density tACS and EEG testing 14 participants and used a very similar experimental procedure to ours, as they had two separate days and three stimulation condition. Another study that used a combination of behavioral, neurostimulation and neuroimaging technique (Saiote et al., 2013; PLOS ONE), investigated BOLD activity induced by high-frequency tRNS during a visuomotor task (10 subjects per condition) and found a behavioral improvement after hf-tRNS and cathodal tDCS and a reduction of learning-related brain activity.

In a different study that used the combination of learning and neurostimulation techniques only, Snowball and colleagues (Snowball et al., *Current Biology: CB*, *23*(11), 987–992) tested 25 subjects altogether across 4 conditions.

Another study conducted by Lewis and colleagues (Lewis et al., PNAS, 2009) investigated the role of spontaneous brain activity for brain dynamics of 14 participants.

Other studies that used a combination of neurostimulation techniques and behavior and/or neuroimaging techniques and used a similar (or smaller) number of participants are reported below.

-Moos et al. (2012). *Journal of Neuroscience*, *32*(46), 16360-16368.

-Polanía, et al. (2012). *Current Biology*, *22*(14), 1314-1318.

-Quartarone, et al. (2004).  NeuroReport, 15(8), 1287- 1291

-Kanai, R., et al. (2008) *Current Biology* *18*(23), 1839–1843

-Wen, et al. (2012). *The Journal of Neuroscience* *32*(4), 1284–1292.

-Cappelletti, et al. (2013)*The Journal of Neuroscience*, *33*(37), 14899–14907.

- Schmidt, et al. (2014). *NeuroImage*, *98*, 216–224.

**Replicates**

- You should report how often each experiment was performed
- You should include a definition of biological versus technical replication
- The data obtained should be provided and sufficient information should be provided to indicate the number of independent biological and/or technical replicates
- If you encountered any outliers, you should describe how these were handled
- Criteria for exclusion/inclusion of data should be clearly stated
- High-throughput sequence data should be uploaded before submission, with a private link for reviewers provided (these are available from both GEO and ArrayExpress)

This is a multi-session learning experiment. Each participant underwent a total of 7 experimental sessions, and the experiment lasted 7days for each subject (1 thresholding day, 1 pre-test day, 1 post-test day, 4 days of training). We had 3 different conditions (one for each stimulation setting) in a between-subjects design. Details are all reported in the “Materials and Methods” section of the paper. We ensured subjects’ performance across the 3 groups did not differ at baseline (pre-test session) and the staircase procedure used to psychophysically measure their threshold performance for both behavioral tasks ensured that any potential variability in performance was accounted for. Also, we embedded catch trials during each session (beside the threshold session) so that we ensured an appropriate level of attention to the task throughout the entire experiment. Outliers were decided on subjects’ performance on catch trials. Seven subjects were excluded due to head motion during one or both scanning procedures or due to inadequate behavioral performance on catch trials. We made the raw data available and they can be found at the following links:

fMRI data: <https://doi.org/10.5281/zenodo.5558975>

Training Data: <https://doi.org/10.5281/zenodo.4634008>

Pre-post data: <https://doi.org/10.5281/zenodo.4621644>

Also, data frames and analysis are available anytime along the reviewing process at the following link: [https://istitutoitalianotecnologia-my.sharepoint.com/federica_conto_iit_it/DataframesExperiment](https://istitutoitalianotecnologia-my.sharepoint.com/:f:/g/personal/federica_conto_iit_it/Ev4QOQ45F2NPuU-lDpozEoIBs1LGaR_yxLVRupVeiQUtkw?e=maErrp)

Please outline where this information can be found within the submission (e.g., sections or figure legends), or explain why this information doesn’t apply to your submission:

**Statistical reporting**

- Statistical analysis methods should be described and justified
- Raw data should be presented in figures whenever informative to do so (typically when N per group is less than 10)
- For each experiment, you should identify the statistical tests used, exact values of N, definitions of center, methods of multiple test correction, and dispersion and precision measures (e.g., mean, median, SD, SEM, confidence intervals; and, for the major substantive results, a measure of effect size (e.g., Pearson's r, Cohen's d)
- Report exact p-values wherever possible alongside the summary statistics and 95% confidence intervals. These should be reported for all key questions and not only when the p-value is less than 0.05.

Please outline where this information can be found within the submission (e.g., sections or figure legends), or explain why this information doesn’t apply to your submission:

The statistical analyses information for both behavioral and MRI data is reported in the manuscript in section ‘Materials and Methods’, subsection ‘Data Analysis’ (analysis for behavioral and fMRI data are described separately). Specifically, in the manuscript we have reported all the statistical tests we used, number of subjects, and the corrections applied for multiple comparison. All statistical values, Mean, SEM etc, are reported in the manuscript (“Results” section, as well as in the Figures description) and all the p-values, including the p-values larger than 0.05, are reported in the text (and not in the figure legends). Figures are also described in the text.

(For large datasets, or papers with a very large number of statistical tests, you may upload a single table file with tests, Ns, etc., with reference to sections in the manuscript.)

**Group allocation**

- Indicate how samples were allocated into experimental groups (in the case of clinical studies, please specify allocation to treatment method); if randomization was used, please also state if restricted randomization was applied
- Indicate if masking was used during group allocation, data collection and/or data analysis

Please outline where this information can be found within the submission (e.g., sections or figure legends), or explain why this information doesn’t apply to your submission:

Subjects were randomly assigned to one of three stimulation conditions. This information is described in the “Materials and Methods” section (subsection ‘Study Design’). Single-blind procedure was used during data collection.

**Additional data files (“source data”)**

- We encourage you to upload relevant additional data files, such as numerical data that are represented as a graph in a figure, or as a summary table
- Where provided, these should be in the most useful format, and they can be uploaded as “Source data” files linked to a main figure or table
- Include model definition files including the full list of parameters used
- Include code used for data analysis (e.g., R, MatLab)
- Avoid stating that data files are “available upon request”

Please indicate the figures or tables for which source data files have been provided:

Summary tables for both behavioral and MRI data used to make the figures are available at the follow links: [https://istitutoitalianotecnologia-my.sharepoint.com/federica_conto_iit_it/tRNSfMRI_Dataframes](https://istitutoitalianotecnologia-my.sharepoint.com/:f:/g/personal/federica_conto_iit_it/Ev4QOQ45F2NPuU-lDpozEoIBs1LGaR_yxLVRupVeiQUtkw?e=maErrp)
